# Supplementary material for: Chromothripsis during telomere crisis is independent of NHEJ, and consistent with a replicative origin
Source: Genome Res. 2019 May;29(5):737–49. doi: 10.1101/gr.240705.118 (PMC6499312; doi:10.1101/gr.240705.118)
Supplement: Supplemental Material [file supp_gr.240705.118_Supplemental_file_1.zip › contigs/annotated_contigs/DB46/contig.2.DB46_length_801_mean_cov_6.50436953808.docx]

**DB46_length_801_mean_cov_6.50436953808**

```
CGAGTGATTTGAATAGGCTATGTTTTACTGGTTTCCTTATATTACGTAAATGTCAGTGGAATACATGAAATGGGGATCATTATAGGTCT
 >chr20:16738576-16738980 + E=3e-228
CAGAACTTCAGGAATTTTTCCACATTGTGAGGAATGATGGCTCAGGCTTCTTTTGTTTGTGGGACCATGTTCAACTAACCACTTGCTTA

CCAATTCACTTATCTAAAAAATGGGTAATGCCTTTGGAGGGTAACTATCTTTGGGATAATTGTGAGCATTAAGTTAGATACTGTATGCA

AAATCACCCAACACACTACACAGTACACGGTTGTATGTAATAAATGGTAGTAAAATTAAACTTTAAAATGAGAGGGTTTTAAAACATTT

TGAAATGTTTGAAGAACTAGACTTCTTTTTAGTGAATTAGAAGTATAT|T|CAAAATGAAATGGAATAACTGAAGTAATTGTTGGAATT
                                                  >chr20:16739846-16740242 + E=2e-217
GTCAGTAGAGTTTTACCAGAAACATTTGGGTTATTGTAGTCACCATCCCCCTACAATCCCTCCTTCCCACTCCTGAACTGCTTTTGTGA

AGACATAGTCTGTGTGAAACATATTTGAGTCACAGATGACCTAGTTCCTTGTTCACAATGAACATTGTGACTAAATTTAGCAGCAGTTT

TTGTGCAGTAAACTTTTAGAACACCATGGCGTATTTCCTAAGCTTAGAAGATGAGAGTGTCATTCTCTCTAGCATCACCCACTTCTGAC

CTCAGATAAATGGGGCCCATCACTGCTTCTAAGGTTTATTGGCTGAGGGGATGTGAATATCCTCAGGGATCAGGTTTAGTACAGGGAAA

TG
```
